# Traditional Chinese medicine as a viable option for managing vascular cognitive impairment: A ray of hope

**DOI:** 10.1097/MD.0000000000041694

**Published:** 2025-03-14

**Authors:** Di Liu, YueYu Zhao, RunFeng Liu, BaoGuang Qiao, XinRu Lu, YuanYuan Bei, Yin Niu, XiaoNi Yang

**Affiliations:** a College of Traditional Chinese Medicine, Shandong Second Medical University, Weifang, China; b Department of Pain, Heze Municipal Hospital, Heze, China; c Department of Traditional Chinese Medicine, Weifang People’s Hospital, Weifang, China; d College of Medical, Shandong Yingcai University, Jinan, China; e Shandong Jiaotong College Hospital, Jinan, China; f Department of Endocrinology, People’s Hospital of Dingtao District, Heze, China; g Department of Traditional Chinese Medicine, The First Affiliated Hospital of Shandong First Medical University and Shandong Provincial Qianfoshan Hospital, Jinan, China.

**Keywords:** pathogenesis, therapy, traditional Chinese medicine, vascular cognitive impairment, vascular dementia

## Abstract

Vascular cognitive impairment (VCI) is a prevalent cognitive disorder resulting from cerebrovascular disease and encompasses a spectrum of cognitive deficits, ranging from mild impairment to vascular dementia (VD). VCI is responsible for a minimum of 20% to 40% of all cases of dementia, with its prevalence ranking second only to Alzheimer’s disease on a global scale. The pathogenesis of VCI is complex and includes a lack of cholinergic nerve cells, inflammation, oxidative stress, alterations in the blood-brain barrier, and cell apoptosis. Current guideline-recommended drugs have unsatisfactory therapeutic effects. However, traditional Chinese medicine (TCM) has long been associated with treating dementia, and numerous studies regarding treating dementia with TCM have been conducted. The etiology and pathogenesis of VaD are linked to deficiencies in the spleen and kidney, as well as phlegm turbidity. Treatment involves benefiting the spleen and kidney, improving blood circulation, removing blood stasis, and dispelling phlegm. Moreover, TCM presents benefits such as few adverse effects, low cost, long-term use suitability, and preventive effects. This review outlines the pathogenesis of VCI in both modern medicine and TCM, examines traditional prescriptions and single-agent ingredients with their pharmacological effects, emphasizes TCM’s unique features, and explores its multi-targeted approach to treating VCI.

## 1. Introduction

The concept of vascular cognitive impairment (VCI) was initially proposed by Hachinski et al,^[1]^ and pertains to the progression from subjective cognitive decline and mild cognitive impairment to complete dementia.^[2]^ The clinical manifestations of VCI are heterogeneous and exhibit varying degrees of severity, typically encompassing deficits in attention, memory, and executive function.^[3]^, Genetic predisposition and environmental risk factors like unhealthy lifestyles, hypertension, cardiovascular disease, and metabolic disorders interact, leading to brain changes that result in VCI.^[4]^ vascular dementia (VD) is the most severe form of VCI.^[5]^ According to Vascular Impairment of Cognition Classification Consensus Study (VICCCS), VaD can be divided into 4 main subtypes: post-stroke dementia (PSD), defined as dementia occurring within 6 months after stroke; subcortical ischemic vascular dementia (SIVaD); multiple infarcts (cortical) dementia; and mixed dementia. VCI has become a crucial factor contributing to neurological disorders and cognitive decline worldwide, particularly among aging adults.^[5]^ VCI accounts for at least 20% of all dementia diagnoses and is the second type of dementia after Alzheimer’s disease (AD). The pathological characteristics of AD include the excessive accumulation of amyloid plaques within brain tissue and the formation of neurofibrillary tangles.^[6]^ Although VD and AD differ in their underlying pathological mechanisms, they mutually facilitate each other’s progression during disease development. Research has demonstrated a positive correlation between vascular stiffness and the onset of AD, as well as an acceleration of its progression.^[7]^ Consequently, it can be inferred that vascular abnormalities significantly contribute to the pathogenesis of both AD and VD. Furthermore, as individuals age, vascular risk factors pose substantial threats to overall health.Epidemiology suggests that the number of people affected globally was approximately 50 million in 2018 and is expected to triple by 2050, with growing population numbers and aging further increasing the healthcare burden on society.^[8]^ The prognosis of VCI is highly correlated with the quality of life for older adults.^[9]^

**Figure 1.**
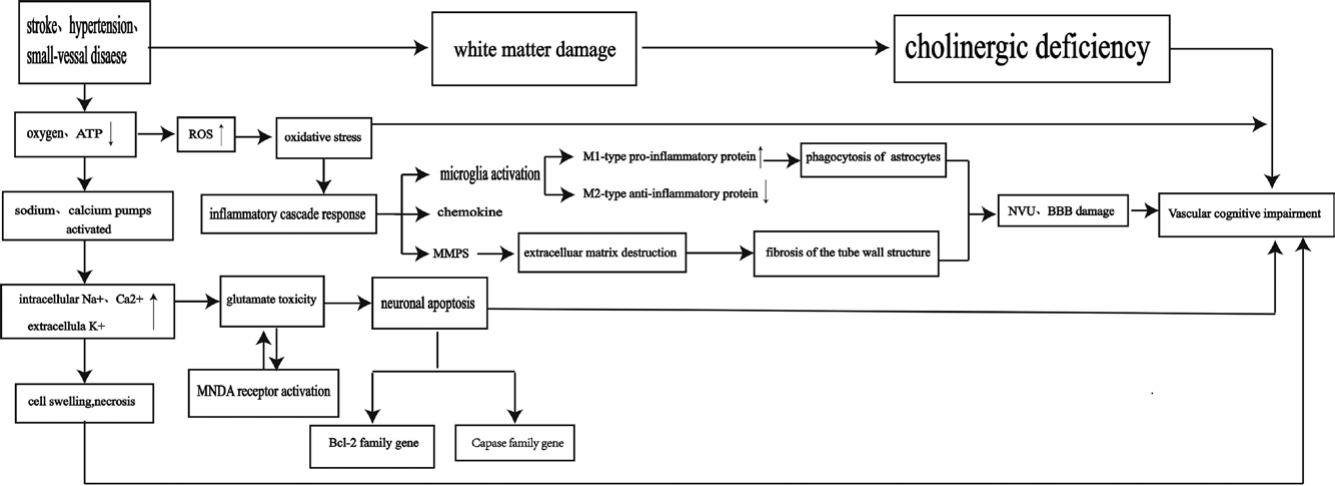
Vascular events in the brain result in ischemia and hypoxia in the tissue, leading to energy depletion and subsequent disruption of intracellular and extracellular ion balance. This disruption triggers oxidative stress, inflammatory cascades, and damage to the blood-brain barrier. Ultimately, this cascade of events culminates in brain cholinergic deficiency and nerve cell apoptosis.

Chronic cerebral hypoperfusion is a common pathophysiological in VCI that often results in neurovascular degeneration, neuronal damage, and blood-brain barrier changes. This state can induce β-amyloid-related oxidative stress, mitochondrial dysfunction, and neuroinflammation, exacerbating the disease. Abnormal neuroinflammation and apoptosis are key factors contributing to endothelial and neuronal damage.^[10]^ Specifically, cerebral ischemia-hypoxia leads to neuronal loss, hippocampal atrophy, and cholinergic deficiency through a series of pathological changes (Fig. 1). Consequently, elucidating these mechanisms is essential for the advancement of efficacious therapeutic interventions for vascular dementia.^[11]^

Two drug classes, cholinesterase inhibitors and NMDA antagonists, are approved for treating cognitive disorders like VD and AD. They alleviate symptoms but do not cure or prevent the diseases.^[12]^ Lecanemab, a novel amyloid-sequestering agent, recently received accelerated Food and Drug Administration approval for the treatment of mild dementia due to AD and mild cognitive impairment (MCI), However, the risk of bleeding and its high price have limited its use.^[13]^ Traditional Chinese medicine (TCM), with a history spanning over 2000 years, serves as an effective complement to conventional treatments for VCI. TCM has the potential to mitigate drug resistance and enhance patients’ quality of life. Chinese herbal medicine, characterized by its affordability, accessibility, and minimal disruption, is widely accepted by patients. Exploring the potential of TCM in addressing dementia may provide novel therapeutic approaches, thereby improving patient quality of life and advancing clinical care for this condition.^[14]^

Under TCM, VCI belongs to the category of “dementia,” and an independent theory of cognitive impairment is presented based on TCM.^[15]^ The pathogenesis of dementia under TCM includes spleen and kidney deficiency, phlegm stagnation, and blood stasis blocking the vessels, and involves a series of organs, including the brain, kidney, heart, liver and spleen.^[16]^ Therefore, the treatment is often based on tonifying the spleen and kidney, promoting blood circulation, removing blood stasis, resolving phlegm, and clearing the orifices.^[17]^ The concept of spleen deficiency and kidney deficiency and phlegm turbidity can be understood as follows: in the theory of TCM, the kidney is the most important organ in the growth and development of the human body. It is the innate root, the source of life, and the material basis for the formation of viscera and body activities.^[18]^ According to Western medicine, the kidney can produce erythropoietin,^[19]^ regulate sodium, potassium, calcium and other electrolytes, maintain the stability of body fluid, and maintain blood pressure.^[20]^ When the kidneys fail, symptoms such as anemia, electrolyte disturbances, blood pressure imbalances, and body swelling can occur. In the theory of TCM, the spleen is the acquired foundation, which mainly transforms the essence of water and grain into seminal blood and body fluid. According to western medicine, the spleen is the largest lymphoid organ in the human body, which has the function of immune response, hematopoiesis, and clearance of red blood cells and platelets.^[21]^ When the spleen fails, the body will have symptoms such as low immunity, repeated infections, loss of appetite, and fatigue.^[22]^ Phlegm is the pathological product of human body’s water metabolism disorder, which is divided into visible phlegm and invisible phlegm in TCM.^[23]^ Invisible phlegm refers to the pathological metabolites of the body. Invisible phlegm is deposited in various zangfu meridians, causing corresponding diseases. Visible sputum is the metabolic waste product that is coughed out of the respiratory tract.^[24]^ In Western medicine, phlegm turbidity is associated with diseases such as chronic inflammation, obesity, and metabolic disorders. Modern medical research is exploring the link between TCM theory and Western medicine, particularly in neurodegenerative diseases like AD. Studies suggest kidney deficiency and phlegm may be involved in these diseases. Notopterygium incisum extract has been shown to improve cognitive deficits in AD mice, potentially by inhibiting amyloid β, tau pathology, and neuroinflammation.^[25]^ This offers a fresh perspective on spleen and kidney deficiencies and phlegm turbidity in TCM, potentially inspiring new treatment approaches. The following chapters summarize the pathogenesis of VCI and introduce the role of related traditional Chinese medicine in VCI.

## 2. Pathological mechanisms of VCI and effective TCM

### 2.1. Cholinergic deficiency in VCI and potentially effective TCM

Acylcholine (ACh) plays a key role in shaping the activity of cortical neurons and supporting cognitive functions such as learning, memory, and attention.^[26]^ It is catalyzed by acetylcholine transferase.^[27]^ Studies show that ACH and catalyzed by acetylcholine transferase are decreased in patients with AD and VCI.^[28]^ Vascular events, such as ischemia and hypoxia, in patients with VCI affect the central cholinergic system of the brain, resulting in cholinergic insufficiency. Executive dysfunction and memory issues have been associated with acetylcholine deficits.^[29]^ However, exogenous ACh supplementation can functionally compensate for the decrease in ACh levels and improve memory and cognitive impairment.^[30]^ Therefore, cholinergic content needs to be adjusted to improve the cholinergic function for the prevention and treatment of VCI.

The basic pathogenesis of VCI is deficiency of kidney essence and obstruction of brain orifices. The kidney is the main storage of essence and marrow, and the brain is the sea of marrow. When the kidney is full of essence qi, the marrow is active, and the marrow sea is nourished; therefore, the brain function is sound. Cholinergic deficiency corresponds to the deficiency of kidney essence; therefore, the TCM for tonifying the kidney can promote the energy metabolism and utilization of central neurons and restore the brain cholinergic function.

Kai Xin San (KXS) originates from the volume of Essential Prescriptions for Urgent Needs (Beiji Qianjin Yaofang), which consists of *Ginseng*, *Polygala*, *Acorus calamus*, and *Poria.* The main active ingredients of KXS are saponins, xanthones, oligosaccharide esters, triterpenoids, volatile oils, and flavonoids. Among them, saponins are the main active ingredients. KXS has been used for thousands of years to treat depression and dementia.^[31]^ Saponins originate from ginseng, which is considered the “king of tonics” owing to its ability to strengthen the body and kidneys, and provide nerve nutrition. Network pharmacology and molecular docking can identify potential targets and pathways through the “component-target-pathway” approach. This meta-analysis revealed that KXS improves the cognitive benefits in AD models by reducing the time of escape latency (SMD = −16.84) as well as increasing the number of cross-platform (SMD = 2.56) and proportion of time in the target quadrant (SMD = 7.52). It was also found that KXS exerted anti-AD effects through cholinergic pathway.^[32]^

### 2.2. Glutamate toxicity in VCI and potentially effective TCM

**Figure 2.**
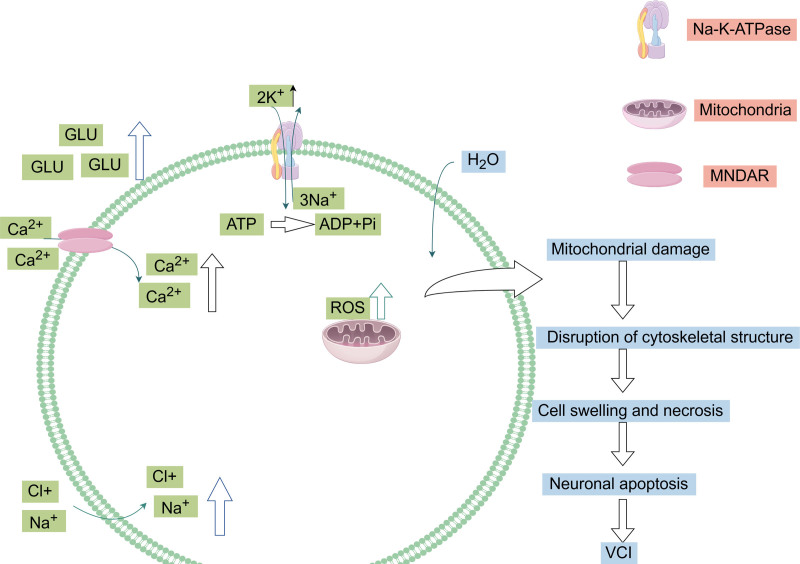
When cellular energy levels are depleted, the activity of Na^+^/K^+^-ATPases and Ca^2+^/H^+^-ATPases at the cell membrane is decreased, resulting in elevated extracellular K^+^ and intracellular Ca^2+^ levels. This imbalance leads to excessive release of extracellular glutamate and a subsequent influx of Ca^2+^, causing calcium overload and excitotoxicity. Concurrently, passive movement of water follows the ion influx, resulting in cytotoxic edema. Intracellular Ca^2+^ overload also triggers the generation of free radicals and the activation of Ca^2+^-dependent enzymes, ultimately disrupting the cell membrane structure and inducing apoptosis in neuronal cells.

Vascular events in the brain result in insufficient supply of blood oxygen, blood glucose, and ATP, which affect the activity of sodium-potassium ATPase, and then affect the sodium and calcium pumps on the cell membrane, leading to ion imbalance, depolarization of the cell membrane, and calcium overload.^[33]^ Calcium overload can cause excessive release of glutamate and trigger excitotoxicity. The accumulation of glutamate can activate calcium-permeable ionic NMDA receptors, which in turn aggravates calcium overload.^[34]^ Excessive glutamate accumulation in the synaptic cleft leads to the loss and death of neurons, causing a cognitive impairment.^[35]^ In this study, we found that the evaluation of glutamate levels can aid in the early diagnosis and classification of cognitive dysfunction.^[36]^ Overstimulation of glutamate receptors can cause excitotoxicity, leading to neurodegenerative diseases.^[37]^ Therefore, inhibition of glutamate toxicity is a potential therapeutic target for VCI (Fig. 2).

Phlegm blocking brain orifices and leading to dementia was formally proposed in the Yuan Dynasty.^[16]^ Chinese medicine categorizes phlegm into tangible and intangible phlegm. Tangible phlegm refers to visible phlegm. Invisible phlegm corresponds to the pathological metabolites of the body. Invisible phlegm deposited in various organs and meridions causes corresponding diseases. Therefore, invisible phlegm in the brain can cause diseases such as vertigo and dementia.^[38]^ The spleen is responsible for the transport and transformation of food and water incorporated into the body.^[39]^ If the spleen is unable to transport the body fluids out in time, the retention of metabolites in the body can lead to phlegm, dampness, or coagulation of organs. The spleen represents the metabolic capacity of the body.^[40]^ Excessive glutamate accumulation in the synaptic cleft is equivalent to the invisible phlegm accumulation in the body. Therefore, the benefits of TCM in tonifying the kidney, strengthening the spleen, clearing phlegm, and opening the orifices can improve the symptoms of cognitive impairment.

Modified Erchen decoction, a classic expectorant prescription, is composed of *Pinelliae rhizoma, Citri reticulatae pericarpium, Poria,* and *Glycyrrhizae radix et rhizome.* Modified Erchen decoction has demonstrated a significant amelioration effect on cognitive dysfunction in VaD rats via JAK2/STAT3 and JNK/BAX signaling pathways.^[41]^ Shenzhi Jiannao formula is composed of *Panax ginseng C.A.Mey.*, *Anemarrhena asphodeloides*, and *Paeoniae Radix Rubra.* This formula promoted PC12 cell proliferation, inhibited glutamate-induced cell death, and reduced hippocampal neuron loss, thereby improving memory.^[42]^ KXS can significantly reduce the glutamate concentration in the brain tissue of rats with vascular dementia. It can also inhibit glutamate neurotoxicity and improve cognitive function mainly by activating the Shh/Ptch1 signaling pathway in rats.^[43]^

### 2.3. Oxidative stress and neuroinflammation in VCI and potential effectiveness of TCM therapy

Oxidative phosphorylation, which occurs in mitochondria, is the main pathway for ATP production. This process is accompanied by the production of free radicals or reactive oxygen species (ROS).^[44]^ Cellular senescence is closely associated with oxidative stress.^[45]^ A state of imbalance between oxidative and antioxidant effects in the body causes oxidative stress. Superoxide dismutase (SOD) and cytochrome c are the main antioxidants. When the increase in ROS under pathological conditions, such as ischemia and hypoxia, exceeds the scavenging capacity of antioxidants, such as SOD, it causes apoptosis or necrosis of neurons and induces cognitive impairment.^[46]^

Neuroinflammation features a primary role in the pathogenesis of vascular cognitive impairment.^[47]^ Hypoxia induces oxidative stress to produce ROS, which stimulates the activation of inflammasomes. Microglia in the nervous system are the main macrophage population in the CNS.^[48]^ As the main innate immune cells of the inflammatory response in the brain, they are fully activated in response to injury, accompanied by the release of a large number of inflammatory mediators, including cytokines, such as interleukin (IL), tumor necrosis factor-alpha, chemokines, and matrix metalloproteinases (MMPs).^[49]^ Under normal conditions, microglia are “scavengers” in neural tissues, which can remove tissue debris and ruptured cells, and have a bi-directional nature in inflammatory responses, i.e., a pro-inflammatory M1 phenotype and an anti-inflammatory M2 phenotype.^[50]^ When the blood vessel is damaged, under the action of chemokines, microglia enter the blood vessel to play a protective role. However, under the continuous inflammatory stimulation, microglia turn from the M2 protective phenotype to the M1 deleterious phenotype. Then, they promote the release of inflammatory factors and phagocytosis of astrocytes, and MMPs cause the destruction of the extracellular matrix, accompanied by the reconstruction of the blood vessel wall, fibrosis, and further damage to the blood–brain barrier (BBB).^[51]^ This eventually leads to cognitive impairment.

Oxidative stress is closely related to inflammatory response. It induces inflammation, which in turn exacerbates oxidative stress.^[52]^ The interplay of oxidative disorders and neuroinflammation promotes the pathogenesis of dementia. Oxidative stress is caused by excessive release of ROS in the brain. ROS can induce the activation of glial cells, stimulate the expression of inflammatory cytokines, and cause chronic neuroinflammation. In turn, constantly activated microglia and astrocytes can produce large amounts of ROS, thereby promoting oxidative stress. Oxidative stress and harmful inflammation form a vicious cycle and cooperate to promote the development of dementia.^[43]^ Therefore, anti-inflammatory and anti-oxidative stress herbs may improve the symptoms of cognitive impairment.

Yin-yang theory is one of the core theories of TCM. Irregular living habits and emotional stimulation affect the function of the body and destroy the health of the body’s qi. When healthy qi is insufficient, the body’s ability to resist external stimuli is attenuated. Insufficient function of the organs, coupled with the reduced function of clearing toxins, causes disease. Therefore, when the body’s oxidation and antioxidant system is out of balance, and the imbalance between M2 anti-inflammatory and M1 pro-inflammatory causes yin-yang imbalance, the body’s positive qi is suppressed by evil qi, causing disease and cognitive impairment.^[53]^ On this basis, anti-inflammatory antioxidant TCM can balance the inflammatory response caused by excessive pro-inflammatory factors, improve oxidative stress,^[53]^ and reduce the symptoms of patients with VCI.

Huanglian-jiedu Decoction (HLJDD) is famous for its heat-clearing and detoxifying properties comprising Rhizoma coptidis, Scutellaria baicalensis, Phellodendri, and Gardenia jasminoides. Because of its powerful anti-inflammatory and anti-oxidation, it is widely used in the treatment of tumors, and intestinal and cerebrovascular diseases among other ailments.^[54]^ Previous studies found that HLJDD can inhibit M1 macrophages polarization and promote M2 macrophages to reduce inflammation, ease the hardening of the arteries, and achieve stable plaques.^[55]^ The formula also alleviated cognitive function in rats by inhibiting the NLRP3 inflammasome signaling pathway.^[56]^ ShenmaYizhi decoction (SMYZD) is a traditional Chinese medicine prescription proven effective for treating vascular dementia, with active ingredients including gastrodin, ferulic acid, ginsenosides, and β-sitosterol. The study found that SMYZD can improve VD and SOD, gsh-px, GSH activities, decrease MDA content, and improve the symptoms of cognitive impairment in rats.^[57]^

### 2.4. Disruption of the BBB

**Figure 3.**
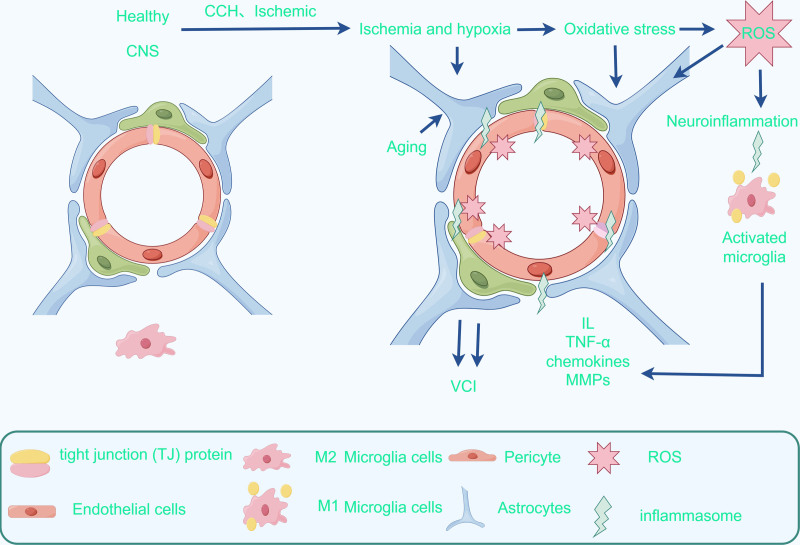
Ischemia triggers a cascade of events where ischemic tissues release inflammatory factors that stimulate the immune response, resulting in the release of additional inflammatory mediators and exacerbating the inflammatory reaction. Concurrently, microglia transition to a pro-inflammatory M1 phenotype, releasing cytotoxic substances that target TJ junction proteins in the blood-brain barrier, ultimately increasing brain permeability and culminating in brain edema and tissue necrosis.

The BBB serves as a protective barrier that separates circulating blood from the parenchymal tissues of the brain. Research indicates that endothelial dysfunction and disruption of the BBB play a role in the pathophysiology of VCI.^[58]^ Anatomically, the BBB consists of capillary endothelial cells, basement membranes, and astrocytes, with tight junction proteins between capillary endothelial cells serving as its primary structural component.^[59]^ The BBB plays a crucial role in regulating the passage of ions and mediators across the vascular endothelial cells. In instances of reduced perfusion to brain tissue, the integrity of tight junction proteins on these cells may be compromised, leading to an increase in BBB permeability. This heightened permeability allows for the infiltration of pathogens and immune molecules into the brain tissue, resulting in potential harm to brain cells and exacerbating cellular damage.^[58]^ The BBB is recognized as an integral component of the neurovascular unit (NVU) system, encompassing neuronal cells, glial cells (microglia, astrocytes, and oligodendrocytes), vascular cells (endothelial cells, pericytes, and vascular smooth muscle cells), and extracellular matrix components.^[60,61]^ The primary role of the NVU is to maintain homeostasis in the central nervous system by regulating cerebral blood flow in response to various stress injuries. This regulation is achieved through the interaction of astrocytes with pericytes and vascular smooth muscle cells, which control vascular contraction and diastole. However, disruptions in the interaction between endothelial cells and nerve cells due to severe vascular damage can impair the function of the NVU, leading to further neuronal damage and cognitive impairment.^[62,63]^ It is conceivable that therapeutic interventions aimed at enhancing the integrity of the BBB and NVU may facilitate the control of VCI through modulation of brain function (Fig. 3).^[58]^

TCM posits that meridians serve as the pathways for the movement of qi and blood.^[64]^ When there is a deficiency in visceral qi and blood, the function of these channels and collaterals is impaired, leading to the accumulation of pathological substances such as phlegm, blood stasis, and endotoxins. The buildup of toxins ultimately results in damage to the brain collaterals, preventing the replenishment of the brain medulla and resulting in the onset of dementia. Yong-Yan Wang’s theory on the detrimental effects of toxins on brain collaterals has significantly influenced the clinical diagnosis and treatment of brain disorders for many years. The collaterals in this context serve as a component of the blood-brain barrier, with their pathological metabolites being identified as toxins.^[65]^

The Bu Yang Huan Wu Decoction is comprised of 7 herbs, namely *Radix Astragali、Radix Angelica Sinensis、Radix Paeoniae Rubra、Pheretima、Semen Persicae、Flos Carthami、Rhizoma Chuanxiong.*Following middle cerebral artery occlusion (MCAO) in rats, Bu Yang Huan Wu Decoction demonstrated the ability to mitigate blood-brain barrier damage. Assessment of cerebral edema using MRI (water diffusion coefficient) revealed a significant reduction in cerebral edema in the group treated with TCM.^[66]^

### 2.5. Neuronal apoptosis in VCI and potential effective TCM

Apoptosis, known as programmed cell death, is a mechanism by which cells undergo autonomous and orderly death under genetic regulation.^[67]^ Increasing research indicates that neuronal apoptosis is the principal pathogenic process in VCI. The regulation of apoptosis is primarily governed by the B lymphocytoma-2 (Bcl-2) protein. Upon examination of family genes related to apoptosis, including cysteine protease (Caspase) family genes, the apoptosis inhibitor gene Bcl-2, and the pro-apoptosis protein gene Bax, it is evident that Caspase-3 plays a crucial role in the apoptosis signal transduction pathway. In instances of cerebrovascular ischemia, the pre-apoptotic protein Bax undergoes conformational changes in response to apoptotic signals, leading to its translocation from the cytoplasm to the outer membrane of the mitochondria. The formation of a protein dimer with the anti-apoptotic protein Bcl-2, regulation of mitochondrial permeability, and release of small molecules, such as cytochrome c, into the cytoplasm are key mechanisms by which this protein induces neuronal apoptosis. Additionally, its interaction with apoptotic protease activating factor leads to the activation of caspase-9 and caspase-3.^[68]^ Inhibition of neuronal apoptosis presents a promising therapeutic strategy for addressing VCI.

The process of apoptosis in TCM can be conceptualized as the obstruction of blood flow, leading to inadequate blood supply to the brain and subsequent apoptosis and necrosis of nerve cells. Herbal remedies that promote blood circulation and alleviate blood stasis can effectively eliminate the blockage, a concept known in TCM as “removing blood stasis and promoting the generation of new blood.” Enhancing blood circulation and alleviating stasis have been shown to induce the expression of pro-angiogenic growth factors, including VEGF and FGF, in vivo, thereby facilitating the proliferation of microvascular endothelial cells and ultimately promoting angiogenesis.

The Tongqiao Huoxue decoction (TQHXD), as described in the Reformations of the Medical Forests (Yi Lin Gai Cuo) by the esteemed late Qing Dynasty physician Wang Qingren, is recognized for its properties in promoting blood circulation, eliminating blood stasis, and enhancing mental clarity. TQHXD has been shown to enhance cellular vitality and suppress cell apoptosis.^[69]^ TQHXD has the potential to greatly enhance cell survival rates, decrease the expression of Bax/Bcl-2, caspase-3 proteins and mRNA, and suppress apoptosis, thereby serving a neuroprotective function.^[70]^ The Tao-Hong-Si-Wu Decoction has been shown to enhance angiogenesis by modulating the expression of vascular endothelial growth factor (VEGF), CD34, bromodeoxyuridine (BrdU), and von Willebrand factor (vWF), leading to improved cognitive function in rats subjected to ischemia-reperfusion injury.^[71]^ Additionally, research has demonstrated that the HLJDD exerts its angiogenic effects through the PI3K/AKT/HIF-1α/VEGF signaling pathway, thereby ameliorating brain tissue damage induced by ischemia and hypoxia.^[72]^ The administration of SMYZD has been shown to enhance VEGF-A expression, stimulate vascular regeneration, and ameliorate symptoms of VCI in rats.^[73]^ Besides, shouwu-yizhu Decoction (SYD), a prescribed medication known for its kidney-tonifying and blood circulation-promoting properties, was found to upregulate miR-210-mediated VEGF expression, thereby activating the Notch pathway to stimulate angiogenesis and enhance cognitive function in rats.^[74]^

### 2.6. Pathogenesis of VCI VERSUS AD

VCI primarily arises from cerebrovascular injury and dysfunction, with events like strokes altering cerebral microcirculation and impacting cognition. Animal studies suggest oxidative stress, neuroinflammation, and microvascular dysfunction contribute to cognitive deficits in VCI. Additionally, impaired clearance of toxins like amyloid beta by blood vessels is a key factor in cognitive decline.^[75]^ In comparison, the pathogenesis of AD is characterized by greater complexity, encompassing chronic inflammation, amyloid plaque formation, and neuronal degeneration. The immune system in individuals with AD frequently exhibits an activated state, with elevated levels of soluble biomarkers in serum correlating with the progression of the disease. Furthermore, alterations in the microbiome composition may influence the pathogenesis of AD, indicating that brain pathology is not the sole determinant.^[76]^

## 3. Pathological mechanisms of VCI in TCM and analysis of TCM prescriptions for VCI

**Figure 4.**
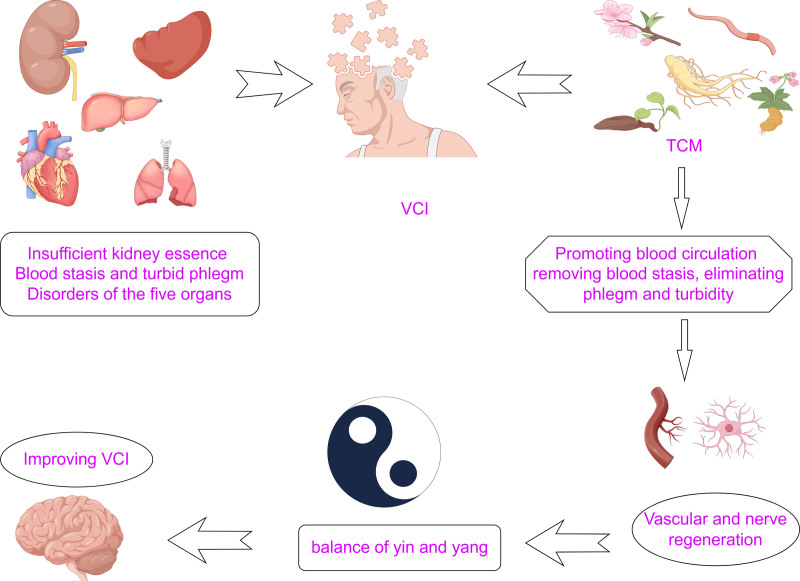
The inadequacy of innate kidney essence, dysfunction of the spleen, and dysfunction of the 5 organs contribute to impaired circulation of qi and blood, resulting in the accumulation of phlegm and stagnation of blood in the collaterals, ultimately leading to dementia. TCM addresses these issues by replenishing kidney essence, promoting blood circulation, eliminating phlegm, and improving cognitive function, ultimately restoring balance between yin and yang and improving VCI. TCM = traditional Chinese medicine, VCI = vascular cognitive impairment.

According to Chinese medicine theory, mental activities are regulated by the proper functioning of qi and blood. Qi facilitates blood circulation and the transportation of nutrients to the organs, as well as the oxygenation of the body, which is essential for maintaining bodily functions. Deficiencies in qi and blood due to weakened internal organs can lead to brain nutritional imbalances, ultimately resulting in dementia.^[16]^ The kidneys are responsible for storing essence, which in turn generates marrow that nourishes the brain, facilitating its normal functioning. The liver functions to regulate and promote the flow of qi throughout the entire body. The diet is mainly transported and absorbed by the spleen to promote the evacuation of body fluid.^[46]^ The underlying cause of this disease lies in the deficiency of kidney essence and qi, with liver stagnation, qi stagnation, and spleen non-circulation exacerbating the transformation of qi and blood, ultimately resulting in qi stagnation and blood stasis. This is further compounded by the accumulation of phlegm and blood stasis, which obstructs the brain collateral. According to TCM theory, the presence of stagnant phlegm in the body can lead to blood stasis in the vessels, ultimately causing damage to arteries and veins. The pathogenesis of this disease can be attributed to a combination of deficiency, phlegm, and blood stasis, with involvement of the kidney, heart, liver, and spleen despite its manifestation in the brain. Many diseases exhibit a combination of deficiency and excess, with deficiency often stemming from a lack of kidney essence and dysfunction of the heart, liver, and spleen, while excess is characterized by congestion, phlegm, and other pathological substances (Fig. 4).^[77]^

Symptoms and prescriptions are fundamental components of TCM, with prescriptions being formulated based on compatibility principles.^[78]^ Compound prescriptions, which are essentially compatible prescriptions, typically exhibit a mode of compatibility known as ‘mutual promotion’.^[79]^ TCM compounds are characterized by their multi-target, multi-component, and multi-mechanism nature, allowing for a coordinated approach through multiple mechanisms to enhance efficacy in the treatment of VCI.^[80]^ TCM emphasizes the importance of syndrome differentiation and treatment. A review of the literature reveals that prescriptions for treating VCI typically fall into 4 categories: tonifying kidney and qi, clearing phlegm and opening orifices, promoting blood circulation and eliminating stasis, and clearing heat and detoxification. The prescriptions were summarized in Table 1.

**Table 1:** Treatment of VCI with TCM prescription

Formula	Ingredients	Possible mechanism	Models	Classification	Ref.
Kai Xin San	*Ginseng, Polygala, Acorus calamus, and Poria*	Antagonized glutamate neurotoxicity through Shh/Ptch1 signaling pathway	MID model injecting homologous blood emboli into the right internal	Clearing phlegm and opening orifices	[31]
Modified Erchen decoction	*Pinelliae rhizoma,Citri reticulatae pericarpium,Poria, Glycyrrhizae radix et rhizoma*	Improving cognitive dysfunction via JAK2/STAT3 and JNK/BAX signaling pathway	2-VO	Clearing phlegm and opening orifices	[41]
Shenzhi Jiannao formula	*Panax ginseng C.A.Mey.Anemarrhena* *Asphodeloides* *Paeoniae Radix Rubra*	Promoting the expression of clathrin-mediated endocytosis and reducing NMDARs-associated glutamate excitotoxicity.	2VO、PC12 cells	Clearing heat and detoxification	[42]
Huanglian-jiedu Decoction	*Rhizoma coptidis, Scutellaria baicalensis, Phellodendri and Gardenia jasminoides*	Inhibited the expression of inflammatory factors and M1 polarization, enhance the M2 polarization	ApoE mice induced by high-fat diet (HFD)	Clearing heat and detoxification	[55]
ShenmaYizhi decoction	*Radix Ginseng, Rhizoma Gastrodiae, Ramuli Euonymi, Rhizoma Chuanxiong*	Activating the AMPK/PPARα/PGC-1α/UCP2 signal pathway to improve mitochondrial structure and energy metabolism	2VO	tonifying kidney and qi	[57]
Bu Yang Huan Wu Decoction	*Radix Astragali、Radix Angelica Sinensis、Radix Paeoniae Rubra、Pheretima、Semen Persicae、Flos Carthami、Rhizoma Chuanxiong*	Inhibiting the activation of the HIF-1 α/VEGF pathway and stabilizing ion channel of β-ENaC in brain	MCAO	tonifying kidney and qi	[66]
Tongqiao Huoxue decoction	*radix paeoniae rubra, rhizoma chuanxiong, peach kernel, carthamus* *tinctorious, and fresh ginger*	blocking the NF-kappa B signaling pathway and down-regulating PTGS2.	MCAO OGD/R-damaged HT22 cell	promoting blood circulation and eliminating stasis,	[69]
Shouwu-yizhi Decoction	*Zhi Shou Wu, Alpinia oxyphylla, Astragalus membranaceus, Gastrodia elata, Salvia miltiorrhiza, Polygala tenuifolia and Ligusticum chuanxiong Hirudo Geosaurus* Ginkgo extract	Increased the expression of miR210, activated the Notch pathway,promoted angiogenesis	MCAO/R	tonifying kidney and qi	[74]
Tao-Hong-Si-Wu Decoction	*Rehmannia glutinosa Libosch., Angelica sinensis(Oliv.) Diels, Paeonia lactiflora Pall,Ligusticum chuanxiong Hort, Prunus persica (L.) Batsch, and Carthamus tinctorius L.,*	Notch signaling was activated to promote the expression of VEGF, CD34, BrdU, and vWF.	MCAO/R	promoting blood circulation and eliminating stasis	[71]

TCM = traditional Chinese medicine, VCI = vascular cognitive impairment.

The generalization of the aforementioned formulas reveals a diverse range of drug effects, including the promotion of vascular endothelial cell proliferation, anti-inflammatory and antioxidant effects. Chinese medicines not only mitigate damage caused by ischemia and hypoxia to brain tissues, but also stimulate vascular neovascularization, enhancing cerebral perfusion and ameliorating cognitive deficits. This highlights the unique advantage of TCM over chemical drugs, which primarily function as agonists or inhibitors. Chinese medicine has the potential to produce synergistic effects among medications, thereby facilitating the regression of the disease.

## 4. Active ingredients of single herb for VCI

**Figure 5.**
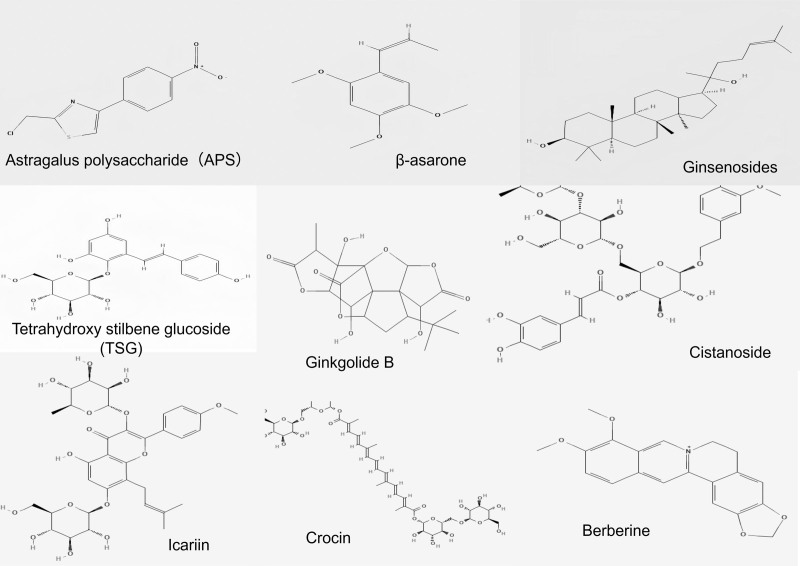
Chemical structures of monomeric herbs.

The chemical composition of monomer Chinese medicines in the treatment of dementia has recently garnered increased attention in academic research. Various studies have illustrated the efficacy of herbal natural products in the prevention of VCI, as detailed in Table 2. The chemical structures of some of the monomeric herbs are summarized in Figure 5.

**Table 2:** Treatment of VCI with active ingredient of monomeric TCMs

Compounds	Sources of TCMs	Function	Molecular mechanisms	Models	Reference
Astragalus polysaccharide (APS)	*Astragalus*	Promoted the recovery of nerve function	Suppression of the Notch1/NFκB pathway	MCAO rat	[81]
**β**-asarone	*Acorus tatarinowii Schott,*	Ameliorated cerebral edema, reduced blood-brain barrier permeability, restored neurological function.	Downregulation of astrocytic NKCC1/AQP4 and JNK/iNOS-mediated ICAM-1/MMP-9 signaling	MCAO rat	[82]
Ginsenosides	*Ginseng*	Improving cognitive impairment in mice by mitigating oxidative stress and inflammation	Increased the expression of BDNF、TrkB	Chronic restraint stress rat	[83]
Tetrahydroxy stilbene glucoside (TSG)	*Polygonum multiflora*	Anti-inflammatory and neural protection	Reduced the level of the M1macrophages, raised the expression of M2 cells	BCCAO murine	[84]
Polygala saponins (PSS)	*Radix Polygalae*	Protected hippocampal neurons against glutamate-induced cytotoxicity	Regulated NMDARs、 reduced glutamate-induced Ca2 + overload	Hippocampal neurons	[85]
Ginkgolide	*Ginkgo biloba*	Reduce neuroinflammation, protect neurons and promote cognitive learning ability	Attenuating inflammatory response via Toll-like Receptor 4 (TLR4)/NF-κB pathway	BCCAO rat、 SH-SY5Y cells	[86]
Cistanoside	*Cistanches*	Promoted angiogenesis、maintained blood-brain barrier integrity	Through the Nrf-2/Keap-1 pathway increased SOD activities	MCAO/ R rat	[87]
Icariin	*Epimedium*	Inhibiting the inflammatory response and achieving neuroprotection	Regulating GPER-ERK-NF-κB signaling and inhibiting microglial activation;Decreased the levels of IL-1β、 TNF-α、Iba1、CD40, CD68 and p-P65-NF-κBand increased the levels of CD206 and p-ERK	MCAO	[88]
Crocin	*Saffron*	Induced the proliferation and migration of neural stem cells, and inhibited the apoptosis of neural stem cells	Activation of Notch signaling pathway and inhibiting the release of inflammatory factors	MCAO/R rat	[89]
Berberine	*Coptis chinensis*	Anti-inflammatory, antioxidant stress	Attenuating TNF-α、IL-1β、MAO release	2-VO rat	[90]

TCM = traditional Chinese medicine, VCI = vascular cognitive impairment.

### 4.1. Astragalus polysaccharide

The compound Astragalus polysaccharide (APS) is an important component derived from the *astragalus* plant.^[91]^ APS, along with flavonoids, saponins, alkaloids, and other compounds, constitutes the main constituents of *astragalus*. APS is recognized as the primary bioactive ingredient in *astragalus*, exhibiting diverse pharmacological properties such as anti-cancer effects, regulation of blood glucose levels, and neuroprotection.^[92]^ Specifically, APS has demonstrated efficacy in mitigating brain damage through the promotion of angiogenesis in studies focused on its protective effects on the central nervous system.^[93]^ The administration of Astragalus injection has been shown to effectively restore the integrity of the blood-brain barrier, reduce escape latency in the Morris water maze task, and enhance cognitive function in mice.^[94]^ Additionally, APS has been demonstrated to facilitate the restoration of sensory and motor function in rats following MCAO, promote neuronal formation, increase neuronal count, and ameliorate symptoms of VCI.^[81]^

### 4.2. β-asarone

β-asarone, a prominent constituent of *Acorus tatarinowii Schott*, plays a significant role in the pathophysiology of neurodegenerative and neurovascular disorders.^[95]^ Research has demonstrated that β-asarone mitigates oxidative stress and amyloid-β deposition in the brain, enhances the levels of brain-derived neurotrophic factor (BDNF), diminishes neuronal apoptosis, and confers neuroprotective effects, ultimately enhancing cognitive function in VCI rats.^[96]^ β-asarone demonstrates a potential therapeutic effect in mitigating cerebral ischemia-induced mitochondrial and synaptic damage, as well as enhancing learning and cognitive abilities in a VD mouse model, likely through modulation of the cAMP/PKA/CREB signaling pathway.^[97]^ Additionally, administration of Acorus calamus extract via intraperitoneal injection post-MCAO in rats was shown to significantly reduce cerebral infarction size, alleviate cerebral edema, decrease blood-brain barrier permeability, restore neurological function, and enhance memory and cognitive function.^[82]^

### 4.3. Ginsenosides

Ginsenosides, extracted from *ginseng*, have demonstrated the ability to modulate synaptic plasticity and the cholinergic system, as well as inhibit the exacerbation of Aβ and tau hyperphosphorylation, neuroinflammation, oxidative stress, and apoptosis.^[98]^ Specifically, Ginsenoside Rb1 has been shown to enhance cholinergic neuron function in the rat brain, reduce oxidative stress and neuroinflammation, and ameliorate memory impairment in rats tested in the Morris water maze.^[99]^ Ginsenosides have been shown to reduce oxidative stress and inflammation through upregulation of PI3K, cAMP response element binding protein (CREB), BDNF, and tyrosine kinase B (TrkB), leading to enhanced cognitive function in mice.^[83]^

### 4.4. Tetrahydroxy stilbene glucoside (TSG)

TSG, the active compound found in *Polygonum multiflora*, has been shown to reverse the polarization state of m1-type microglia and inhibit neuroinflammation via the cGAS-STING signaling pathway, thereby preventing cognitive impairment.^[100]^ The combined administration of *Polygonum multiflora* and *Acorus tatarinosus* was found to enhance cognitive function by elevating acetylcholine levels and suppressing acetylcholinesterase protein expression. It is postulated that compounds such as TSG, emodin, and α-asarone contribute to this effect.^[101]^ In the context of ischemic stroke in mice, TSG demonstrated efficacy in reducing the expression of inflammatory cytokines, inhibiting the nuclear translocation of NF-κB p65, and decreasing ROS production. These effects led to significant anti-inflammatory properties that alleviated symptoms of VCI.^[38]^ Additionally, TSG was found to modulate the balance of M1 and M2 macrophages, exerting anti-inflammatory and neuroprotective effects.^[84]^

### 4.5. Polygala saponins (PSS)

PSS is a significant bioactive constituent found in *Radix Polygalae*, known for its neuroprotective properties including memory enhancement and cognitive improvement.^[102]^ The inhibition of NLRP3 inflammatory vesicles by PSS through shp-2-mediated mitochondrial autophagy results in the removal of Aβ aberrant proteins and the prevention of neuronal death, ultimately leading to enhanced cognitive function.^[103]^ PSS exhibits various neuroprotective properties, including anti-aβ aggregation, anti-inflammation, anti-oxidation, anti-neuronal poptosis, enhancement of central cholinergic system, promotion of neuronal proliferation, and improvement of VCI healing.^[104]^ Furthermore, PSS has been shown to inhibit glutamate-induced neuronal death in the hippocampus and reduce glutamate-induced Ca2 + overload by blocking the activation of NMDA receptors. These results indicate that PSS may protect cultured hippocampal neurons from glutamate-induced cytotoxicity through the modulation of NMDARs.^[85]^

### 4.6. Ginkgolide

Ginkgolide, an active compound derived from *Ginkgo biloba*, exhibits anti-inflammatory, anti-atherosclerotic, and neuroprotective properties. The primary active components of Ginkgo biloba include flavonoids (such as quercetin, kaolin, and isorhamnetin), terpenoids, and ginkgolides (specifically ginkgolides A, B, C, and J).^[105,106]^ In the present study, Liu et al examined the impact of ginkgolide on synaptic plasticity within the rat hippocampus. Their findings revealed that ginkgolide mitigated the long-term potentiation (LTP) induced by θ burst stimulation in CA1 pyramidal neurons of the rat hippocampus. Furthermore, elevated concentrations of ginkgolide hindered the evoked excitatory postsynaptic potentials.^[107]^ Ginkgolide B has been shown to enhance the proliferation, migration, and tube-forming capacity of mouse cerebral vascular endothelial cells and HUVEC, while also inhibiting creatine kinase B (CKB) activity. This inhibition promotes angiogenesis through the CCT/trici-sk1 axis, ultimately leading to improved cognitive deficits and neuroprotective effects following a stroke.^[108]^ Additionally, Ginkgolide B has been found to reduce neuroinflammatory responses by modulating the nuclear factor-κB (NF-κB) signaling pathway, resulting in significant improvements in learning and memory in VD rats.^[86]^

### 4.7. Cistanoside

Cistanoside, a phenylethanoid glycoside compound derived from *Cistanches Herba*, exhibits potent neuroprotective properties.^[109]^ Through mechanisms involving the Nrf-2/Keap-1 pathway, cistanoside has been shown to reduce intracerebral MDA levels, enhance antioxidant activities (SOD, CAT, and GSH-Px), stimulate angiogenesis and neural remodeling, preserve blood-brain barrier integrity, and alleviate symptoms of VCI in a rat model of MCAO/R injury.^[87]^ Cistanoside enhances the proliferation of endogenous neural stem cells by activating the Wnt/β-catenin signaling pathway, ultimately improving cognitive impairments following a stroke.^[110]^

### 4.8. Icariin (ICA)

ICA serves as the primary active component of *Epimedium*,^[111]^ exhibiting the ability to inhibit microglia activation and M1 polarization while promoting M2 polarization through the regulation of the GPER-ERK-NF-κB signaling pathway. This mechanism ultimately suppresses inflammatory responses, making ICA a neuroprotective agent that can alleviate cognitive impairments following an ischemic stroke.^[88]^ Furthermore, ICA demonstrates the capacity to mitigate apoptosis, reduce intracellular and mitochondrial levels of ROS, downregulate the expression of Bax and cleaved-caspase-3, and upregulate the expression of Bcl-2. Additionally, it has been demonstrated that ICA functions as a neuroprotective agent against oxidative stress-induced injury by modulating the Nrf2/Keap1 signaling pathway, and mitigates cognitive deficits following cerebral ischemia and hypoxia.^[112]^ Similarly, ICA has been found to deactivate NMDAR via the ERK/DAPK1 pathway, reduce glutamate-induced excitotoxicity, and exhibit neuroprotective properties in the brain, thereby alleviating post-stroke cognitive impairment.^[113]^ These findings suggest that ICA may hold promise as a therapeutic intervention for VCI.

### 4.9. Crocin

Crocin, a bioactive compound derived from *Saffron*, has been demonstrated to improve cognitive function, alleviate depressive symptoms, and combat neurodegenerative disorders.^[114,115]^ Additionally, Saffronin has been shown to suppress the release of inflammatory cytokines and the activation of microglia and astrocytes by targeting NLRP3 inflammasomes and TLR4 signaling pathways, leading to improved performance in cognitive tasks such as the Morris water maze test in mice.^[116]^ Subsequent examination demonstrated that crocin mitigated malathion-induced neurological alterations and cognitive dysfunction by suppressing TAU hyperphosphorylation and exerting anti-apoptotic effects.^[117]^ Additionally, research has indicated that crocin can modulate the Notch signaling pathway, thereby enhancing the proliferation and migration of neural stem cells and inhibiting their apoptosis in rats following MCAO/R injury.^[89]^ Crocin has been shown to inhibit autophagy and decrease oxidative stress levels by activating the PI3K/Akt/mTOR pathway, thereby exerting a neuroprotective effect and enhancing cognitive function.^[118]^

### 4.10. Berberine

Berberine, an isoquinoline alkaloid derived from *Coptis chinensis* and other Berberis plants, is a natural remedy with diverse advantageous biological properties.^[119,120]^ It has been historically utilized for the treatment of diabetes and gastrointestinal disorders, as well as for its anti-aging properties. The anti-aging effects of berberine are believed to be mediated through the regulation of p16 and cyclin protein expression.^[121]^ Berberine, a small molecule capable of crossing the BBB, has demonstrated potential in the treatment of dementia diseases by reducing cell oxidative stress injury and improving spatial learning and memory abilities in rats through the attenuation of tumor necrosis factor-alpha, IL-1β, and MAO release.^[90,122]^ Recent research suggests that berberine may serve as a promising cholinesterase inhibitor for the treatment of neurodegenerative diseases.^[123]^ Berberine administration in mice with cognitive impairment induced by intracerebral hemorrhage was found to improve cognitive function and alleviate blood-brain barrier disruption. This effect is attributed to the inhibition of inflammatory response and activation of the AMPK/PGC1α signaling pathway.^[124]^

## 5. Conclusion

VCI, like AD, is a neurodegenerative disease characterized by progressive memory decline and cognitive loss.^[125]^ With the advent of the aging society, the incidence of cognitive impairment is gradually increasing, and will become the main cause of death and disability in the elderly.^[15]^ Although cholinesterase inhibitors and NMDA antagonists,^[126]^ as well as the emerging targeted amyloid scavenger Lecanemab,^[127]^ have brought new hope for countless patients with dementia, severe adverse effects and high price often make patients do not have satisfactory treatment results. In recent years, TCM has been widely used in clinical practice in China, especially after Tu Youyou won the Nobel Prize with her invention of anti-malaria medicine Artemisinin (Qinghaosu), which has made TCM to the world and made the application of TCM more international.^[128]^ On October, 2018, the World Health Organization (WHO) included TCM in its globally influential medical guidelines for the first time, which once again elevated the status of TCM in the world’s medical industry.^[129]^ Nowadays, more and more attention has been paid to the scientific application of TCM, such as high throughput screening, proteomics, transcriptomics and other technologies, which can comprehensively interpret the mechanism of TCM from the perspective of modern biology and medicine.^[130]^ Current studies have found that amyloid protein, apoptosis, autophagy, neuroinflammation, glutamate toxicity, oxidative stress, and mitochondrial dysfunction are all involved in the pathogenesis of VCI. The Chinese herbal medicine monomers and extracts mentioned in this paper can improve cognitive function mainly by reducing Aβ deposition and neurofibrillary tangles, regulating central cholinergic and other neurotransmitter abnormalities, increasing cerebral perfusion, protecting cerebral blood vessels, improving cerebral microcirculation, improving mitochondrial function of hippocampal neurons, and preventing hippocampal neuron apoptosis. For example, TSG, a resveratrol analogue stilbene glucoside isolated from polygonum multiflorum Thunb, has anti-inflammatory, anti-oxidation and neuroprotective effects.^[84]^ Berberine is the major active isoquinoline alkaloid of Rhizoma coptidis, a commonly used TCM for the treatment of diabetes and inflammation.^[131]^ Recent experimental evidence suggests its potential to treat various dements, including AD^[132]^、VD and diabetes-related dementia.^[133]^ PSS can Regulate NMDARs、reduce glutamate-induced Ca2 + overload. Cistanoside.^[87]^ There is no denying the great contribution of TCM to dementia.

Meanwhile, due to the diverse etiology and complex pathogenesis of VCI, animal models cannot fully reproduce the pathological changes of human VCI, which makes the evaluation of experimental standards difficult. In recent years, there are more and more clinical studies on the treatment of VCI with TCM.^[134]^ However, the observation period in clinical studies is relatively brief, the sample size is limited, and there is a paucity of large-scale, long-term follow-up clinical evidence. Given that the progression of VCI is a gradual and protracted process, the clinical efficacy of TCM in treating VCI warrants further evaluation. Furthermore, due to the unique characteristics of TCM theory, the dosage and modes of administration of TCM often lack standardization, which raises questions regarding the clinical relevance of research findings. Finally, the urgent need for quality control and standardization of Chinese herbal medicines must be addressed. The presence of numerous unvalidated TCM products on the market poses potential safety risks to patients. Consequently, the development of a rigorous quality control system and standardized clinical application guidelines is essential for enhancing the clinical efficacy of TCM.

In conclusion, despite the numerous challenges that persist in the current treatment of VCI, future advancements in the understanding of its pathological mechanisms and the development of novel therapeutic approaches are anticipated to yield more effective interventions for these prevalent cognitive disorders.

## Author contributions

**Conceptualization:** RunFeng Liu.

**Investigation:** YueYu Zhao, BaoGuang Qiao, XinRu Lu, YuanYuan Bei.

**Supervision:** Yin Niu, XiaoNi Yang.

**Visualization:** YueYu Zhao, BaoGuang Qiao, XinRu Lu, YuanYuan Bei.

**Writing – original draft:** Di Liu, RunFeng Liu.

**Writing – review & editing:** Yin Niu, XiaoNi Yang.
